# Cognitive Functioning and Associated Factors in Older Adults: Results from the Indonesian Family Life Survey-5 (IFLS-5) in 2014-2015

**DOI:** 10.1155/2019/4527647

**Published:** 2019-02-03

**Authors:** Supa Pengpid, Karl Peltzer, Indri Hapsari Susilowati

**Affiliations:** ^1^ASEAN Institute for Health Development, Mahidol University, Salaya, Thailand; ^2^Department of Research & Innovation, University of Limpopo, Turfloop, South Africa; ^3^Department for Management of Science and Technology Development, Ton Duc Thang University, Ho Chi Minh City, Vietnam; ^4^Faculty of Pharmacy, Ton Duc Thang University, Ho Chi Minh City, Vietnam; ^5^Faculty of Public Health, University of Indonesia, Depok, Indonesia

## Abstract

**Objective:**

The study aims to investigate cognitive functioning and associated factors in a national general population-based sample of older Indonesians.

**Methods:**

Participants were 1228 older adults, 65 years and older (median age 70.0 years, Interquartile Range=6.0), who took part in the cross-sectional Indonesian Family Life Survey-5 (IFLS-5) in 2014-15. They were requested to provide information about sociodemographic and various health variables, including cognitive functioning measured with items from the Telephone Survey of Cognitive Status (TICS). Multivariable linear regression analysis was performed to assess the association of sociodemographic factors, health variables, and cognitive functioning.

**Results:**

The overall mean cognition score was 14.7 (SD=4.3) (range 0-34). In adjusted linear regression analysis, older age, having hypertension, and being underweight were negatively associated with better cognitive functioning and higher education was positively associated with better cognitive functioning.

**Conclusion:**

Several sociodemographic and health risk factors for poor cognitive functioning were identified which can guide intervention strategies in Indonesia.

## 1. Introduction

Cognitive function is known to be influenced by many factors such as home environment in childhood, genes, and sociodemographic factors [1]. Cognitive decline is commonly detected at middle age, and from that point onwards age-related decline is the rule [2]. There is little research on cognitive functioning in the general population among older adults in Indonesia [3–5].

Previous studies found that cognitive performance in older adults has been associated with socioeconomic factors, illness conditions and health status, social capital, and health behaviours [6]. Socioeconomic factors for better cognitive functioning include younger age [6–9], female or male sex [7], higher education [4–7, 10], higher economic status [4, 8, 10], and residing in urban areas [8]. Illness conditions and health status factors may include no depressive symptoms [11], no insomnia [12–14], no hypertension [15], no heart failure [16], being not undernourished [17], better self-rated health status [6], higher life satisfaction, and better Quality of Life [6, 18, 19], and not having functional disability [16]. Some studies found that social cohesion or social capital [3, 10, 20] was associated with better cognitive capacity. Several healthy behaviours such as not smoking [21, 22] and physical activity or exercise [6, 23–25] have also been identified as positively linked to cognitive functioning.

The study aims to investigate cognitive functioning and associated factors in a national probability sample of older persons (50 years and above) who participated in the Indonesian Family Life Survey-5 (IFLS-5) in 2014-2015.

## 2. Method

### 2.1. Study Design and Participants

Data were analysed cross-sectionally from the “Indonesian Family Life Survey-5 (IFLS-5)” in 2014-2015 [26]. The IFLS-5 used a multistage stratified sampling design [26]. We followed the methods of Peltzer et al., 2018 [27]. In all, 1228 65 years and older individuals were included with complete cognitive functioning measurements. The response rate was above 90%.

### 2.2. Measures

*Cognitive functioning* was measured with items from the “Telephone Survey of Cognitive Status (TICS)” [28], which was face-to-face interview administered in this study. The TICS included items on awareness of the date and day of the week and a self-reported memory question, with response options of excellent, very good, good, fair, and poor. Then the participant was asked to serially subtract 7s from 100. Then an immediate and delayed word recall of 10 nouns was given [26]. Total scores ranged from 0 to 34. Possible dementia was defined as having scores of 0-8, similar to the modified TICS in the Health and Retirement Study in USA [29].

*Sociodemographic factor* questions included age, gender, education, residential status, country region, and subjective socioeconomic status [26].

*Social capital *was assessed with 4 items, related to the past 12-month participation in “(1) Community meeting, (2) Voluntary labour, (3), Programme to improve the village/neighbourhood, and (4) Religious activities.” Response options, were “yes” or “no” [26] (Cronbach's alpha 0.69). Those who scored 0 times with “yes” were considered as having low social capital.

*Self-reported health status *was measured with the question, “In general, how is our health?” Response options were 1=very healthy, 2=somewhat healthy, 3=somewhat unhealthy, and 4=unhealthy [26].

*Life satisfaction* was assessed with the question, “Please, think about your life as a whole. How satisfied are you with it?” Response options ranged from 1=completely satisfied to 5=not all satisfied [26]. Low life satisfaction was defined as not very or not at all satisfied.

*Chronic medical condition* was assessed with the question, “Has a doctor/paramedic/nurse/midwife ever told you that you had…? High blood pressure, Stroke,” (yes, no) [26]. Hypertension was measured and classified using standard procedures [26, 30].

The* Centres for Epidemiologic Studies Depression Scale (CES-D: *10 items) was used to assess depressive symptoms, and scores 10 or more were classified as having depressive symptoms [31] (Cronbach's alpha = 0.67).

*Insomnia symptoms *were assessed with five items from the “Patient-Reported Outcomes Measurement Information System (PROMIS)” sleep disturbance measure [32] and with five items from the PROMIS sleep impairment measure [33]. Cronbach's alpha was 0.82 for the 10-item scale in this study. Insomnia was classified using criteria close to the “Insomnia Severity Index” [34], with clinically significant insomnia having total scores of ≥21-40.

*Nutrition status*: Heights and weights were taken using standard procedures [26]. Body mass index (BMI) was calculated as weight in kg divided by height in metre squared and underweight classified as <18.5 kg/m^2^ [35].

*Tobacco use *was assessed with two questions: (1) “Have you ever chewed tobacco, smoked a pipe, smoked self-enrolled cigarettes, or smoked cigarettes/cigars?” (yes, no); (2) “Do you still have the habit or have you totally quit?”(still have, quit) [26]. Responses were grouped into never, quitters, and current tobacco users.

*Physical activity* was assessed with an abbreviated version of the “International Physical Activity Questionnaire (IPAQ) short version, for the last 7 days (IPAQ-S7S)” [36]. Physical activity was categorized according to the IPAQ scoring protocol [37] as low, moderate, and high physical activity.

*Functional disability* was assessed by 5 items of Activity of Daily Living (ADL) (Cronbach alpha 0.84) and 6 items of Instrumental Activity of Daily Living (IADL) (Cronbach alpha 0.91) [38, 39]. A total functional disability score was calculated, with having no difficulty=0, one=1, or two or more ADL/IADL items=2.

### 2.3. Data Analysis

Bivariate associations between independent variables and the dependent variable (overall cognition score and possible dementia) were evaluated with linear regression and logistic regression. Variables associated with overall cognition and possible dementia at P<0.05 were subsequently included in a multivariable linear and logistic regression model, respectively. Potential multicollinearity between variables was assessed with variance inflation factors, none of which exceeded critical value.* P* < 0.05 was considered significant. Cross section analysis weights were applied to make the IFLS sample representative of the 2014 Indonesian population in the study provinces [26]. Both the 95% confidence intervals and P values were adjusted taking the survey design of the study into account. All analyses were performed using STATA software version 13.0 (Stata Corporation, College Station, TX, USA).

## 3. Results

### 3.1. Sample Characteristics and Cognitive Functioning

The total sample included 1228 older adults, 65 years and older (median age 70.0 years, Interquartile Range=6.0, age range of 65-101 years) in Indonesia. The proportion of women was 44.7%, 74.2% had no or elementary education, 38.9% described themselves as having medium economic status, 54.1% resided in urban areas, and 60.1% were in Java. More than one in three of the participants (36.0%) rated their health status as unhealthy, 17.8% had low life satisfaction, and 18.3% had low social capital. Two-thirds of the sample (66.1%) had hypertension, 2.9% have had a stroke, 5.3% had depressive symptoms, 10.3% had insomnia symptoms, and 19.1% were underweight. More than one-third of the participants (34.6%) were current tobacco users, 51.1% were physically inactive, and 32.1% had one or more functional disability.

The overall mean cognition score was 14.7 (SD=4.3) (range 0-34). In bivariate analysis, older age, female sex, lower education, lower economic background, rural residence, unhealthy subjective health status, low life satisfaction, depressive symptoms, insomnia symptoms, having underweight, having hypertension, current tobacco use, and functional disability were associated with poorer cognitive functioning (see Table 1).

### 3.2. Predictors of Cognitive Functioning

In adjusted linear regression analysis, older age, having hypertension, and having underweight were negatively associated with better cognitive functioning and higher education was positively associated with better cognitive functioning (see Table 2).

### 3.3. Prevalence and Predictors of Possible Dementia

The prevalence of possible dementia was 6.8%. In multivariable logistic regression analysis, older age, lower education, and residing in Java were associated with possible dementia (see Table 3).

## 4. Discussion

The study aimed to assess cognitive function and its correlates in older adults in Indonesia. The prevalence of possible dementia was 6.8%, which is a little lower than in the American Health and Retirement Study of 2012 (8.8%) [29]. Consistent with previous studies [6–8, 10], this study found that younger age, higher education, and in bivariate analysis better economic status and living in urban areas were associated with better cognitive functioning. The strong association between higher educational level and better cognitive functioning may be related to underperforming of illiterate participants on tasks requiring immediate verbal attention and working memory [40, 41]. The most robust finding was the association of TICS performance with age and education in the multivariable regression models.

In agreement with previous studies [6, 11–14, 18, 19], this study found in bivariate analysis a negative association between depressive symptoms, low life satisfaction, insomnia symptoms, and cognitive functioning. Consistent with previous studies [15, 17], this study found that having hypertension and underweight were associated with poor cognitive functioning.

Unlike some previous studies [6, 8, 16], this study did not find an association between heart failure, self-rated health status, functional disability, and cognitive functioning. Some studies [3, 10, 20] found a positive association between social capital and better cognitive functioning, while this study did not find such an association. Consistent with previous studies [21, 22], this study found in bivariate analysis that current tobacco use was negatively associated with cognitive functioning, while no association was found for physical activity, as found previously [6, 23–25].

### 4.1. Limitations of the Study

The study was limited by the self-reported measurements and the cross-sectional nature of the study.

## 5. Conclusion

Several sociodemographic and health risk factors such as being underweight and having hypertension were identified for poor cognitive functioning that can guide intervention strategies in Indonesia.

## Figures and Tables

**Table 1:** Sample characteristics and prevalence of cognitive functioning among older adults in Indonesia.

Variables		Total sample	Overall cognition score	Bivariate analysis
		N (%)	M (SD)	Beta (95% CI)
All		1228	14.7 (4.3)	
Age in years	65-74	995 (81.2)	15.2 (4.3)	Reference
	75 and over	233 (18.8)	12.6 (3.6)	-2.61 (-3.18 to -2.05)*∗∗∗*
Gender	Female	540 (44.7)	14.4 (4.4)	Reference
	Male	688 (55.3)	15.0 (4.2)	0.54 (0.003 to 1.09)*∗*
Formal education	Low	880 (74.2)	13.8 (4.0)	Reference
	High	346 (25.8)	17.6 (4.0)	3.80 (3.25 to 4.35)*∗∗∗*
Economic background	Poor	408 (34.9)	13.9 (4.0)	Reference
	Medium	492 (38.9)	15.2 (4.4)	1.26 (0.65 to 1.87)*∗∗∗*
	Rich	328 (26.1)	15.2 (4.5)	1.29 (0.60 to 1.97)*∗∗∗*
Residence	Rural	520 (45.9)	13.9 (4.1)	Reference
	Urban	708 (54.1)	15.4 (4.4)	1.49 (0.95 to 2.02)*∗∗∗*
Region	Sumatra	248 (20.2)	14.7 (3.8)	Reference
	Java	738 (60.1)	14.8 (4.4)	0.15 (-0.42 to 0.73)
	Major island groups	242 (19.7)	14.2 (4.2)	-0.41 (-1.12 to 0.30)
Social capital	High	986 (81.7)	14.8 (4.3)	Reference
	Low	242 (18.3)	14.3 (4.3)	-0.52 (-0.15 to 1.19)
Subjective health status	Healthy	762 (64.0)	15.0 (4.4)	Reference
	Unhealthy	466 (36.0)	14.3 (4.2)	-0.68 (-1.23 to -0.13)*∗*
Life satisfaction	Moderate, high	1007 (82.2)	15.0 (4.4)	Reference
	Low	220 (17.8)	13.8 (3.9)	-1.20 (-1.85 to -0.56)*∗∗∗*
Hypertension	No	414 (33.9)	15.3 (4.3)	Reference
	Yes	790 (66.1)	14.5 (4.3)	-0.85 (-1.42 to -0.28)*∗∗*
Stroke	No	1191 (97.1)	14.7 (4.3)	Reference
	Yes	37 (2.9)	14.8 (3.7)	0.11 (-1.20 to 1.43)
Depressive symptoms (≥15 scores)	No	1168 (94.7)	14.8 (4.3)	Reference
	Yes	60 (5.3)	13.6 (3.9)	-1.23 (-2.38 to -0.08)*∗*
Insomnia symptoms	No	1106 (89.7)	14.8 (4.4)	Reference
	Yes	121 (10.3)	14.0 (3.9)	-0.87 (-1.66 to -0.07)*∗*
Underweight (BMI <18.5)	No	981 (80.9)	15.0 (4.4)	Reference
	Yes	234 (19.1)	13.8 (4.1)	-1.16 (-1.82 to -0.49)*∗∗∗*
Tobacco use status	Never, former	812 (65.4)	15.0 (4.4)	Reference
	Current	416 (34.6)	14.3 (4.2)	-0.74 (-1.29 to -0.18)*∗∗*
Physical activity	Medium, high	612 (48.9)	14.9 (4.4)	Reference
	Low	616 (51.1)	14.6 (4.3)	-0.28 (-0.82 to 0.25)
ADL/IADL	None	822 (67.9)	15.0 (4.3)	Reference
	One	301 (23.9)	14.6 (4.3)	-0.69 (-1.30 to -0.07)*∗*
	Two or more	105 (8.2)	13.5 (4.2)	-1.16 (-2.18 to -0.15)*∗*

*∗∗∗*P<0.001; *∗∗*P<0.01; *∗*P<0.05. (I) ADL=(Instrumental) Activities of Daily Living.

**Table 2:** Multivariable linear regression analysis of factors in bivariate analysis associated with overall cognitive scores among older adults in Indonesia.

Variables	Beta (95% CI)	P-value
Age in years		
65-74	Reference	
75 and over	-2.26 (-2.82 to -1.70)	<0.001
Formal education		
Low	Reference	
High	3.28 (2.71 to 3.85)	<0.001
Economic background		
Poor	Reference	
Medium	0.57 (-0.006 to 1.15)	0.053
Rich	0.34 (-0.31 to 0.98)	0.310
Residence		
Rural	Reference	
Urban	0.36 (-0.16 to 0.87)	<0.167
Subjective health status		
Healthy	Reference	
Unhealthy	-0.18 (-0.70 to 0.34)	0.501
Life satisfaction		
Moderate, high	Reference	
Low	-0.16 (-0.84 to 0.51)	0.635
Hypertension		
No	Reference	
Yes	-0.63 (-1.14 to -1.10)	0.018
Depressive symptoms (≥15 scores)		
No	Reference	
Yes	-0.33 (-1.55 to 0.89)	0.594
Insomnia symptoms		
No	Reference	
Yes	0.02 (-0.78 to 0.81)	0.965
Underweight (BMI <18.5)		
No	Reference	
Yes	-0.67 (-1.30 to -0.03)	0.040
Tobacco use status		
Never, former	Reference	
Current	-0.42 (-0.99 to 0.16)	0.158
Functional disability		
ADL/IADL=0	Reference	
ADL/IADL=1	-0.11 (-0.68 to 0.45)	0.698
ADL/IADL=2 or more	-0.29 (-1.26 to 0.69)	0.563

(I) ADL=(Instrumental) Activities of Daily Living.

**Table 3:** Prevalence of possible dementia and bivariate and multivariable logistic regression with prevalence of possible dementia among older adults in Indonesia.

Variables		Possible dementia	Unadjusted Odds Ratio	Adjusted Odds Ratio
		N (%)	(95% CI)	(95% CI)
All		76 (6.8)		
Age in years	65-74	48 (5.4)	1 (Reference)	1 (Reference)
	75 and over	28 (12.9)	2.61 (1.53, 4.46)*∗∗∗*	2.40 (1.37, 4.18)*∗∗*
Gender	Female	39 (7.8)	1 (Reference)	-* *-* *-
	Male	37 (6.0)	0.76 (0.46, 1.27)	
Formal education	Low	72 (8.8)	1 (Reference)	1 (Reference)
	High	4 (0.9)	0.11 (0.03, 0.34)*∗∗∗*	0.14 (0.04, 0.44)*∗∗∗*
Economic background	Poor	30 (7.4)	1 (Reference)	-* *-* *-
	Medium	28 (6.5)	0.86 (0.48, 1.52)	
	Rich	18 (6.5)	0.87 (0.45, 1.68)	
Residence	Rural	44 (9.5)	1 (Reference)	1 (Reference)
	Urban	32 (4.6)	0.46 (0.28, 0.77)*∗∗*	0.62 (0.36, 1.05)
Region	Sumatra	8 (3.1)	1 (Reference)	1 (Reference)
	Java	51 (7.3)	2.42 (1.12, 5.25)*∗*	2.52 (1.14, 5.55)*∗*
	Major island groups	17 (6.9)	2.40 (1.00, 5.75)	2.28 (0.93, 5.59
Social capital	High	51 (6.4)	1 (Reference)	-* *-* *-
	Low	25 (8.6)	1.36 (0.77, 2.39)	
Subjective health status	Healthy	44 (6.2)	1 (Reference)	-* *-* *-
	Unhealthy	32 (7.9)	1.30 (0.78, 2.18)	
Life satisfaction	Moderate, high	62 (6.7)	1 (Reference)	-* *-* *-
	Low	14 (7.5)	1.16 (0.61, 2.22)	
Hypertension	No	18 (4.9)	1 (Reference)	-* *-* *-
	Yes	58 (8.0)	1.73 (0.96, 3.11)	
Stroke	No	76 (7.0)	1 (Reference)	-* *-* *-
	Yes	0 (0.0)	0.08 (0.06, 0.10)*∗∗∗*	
Depressive symptoms (≥15 scores)	No	71 (6.6)	1 (Reference)	-* *-* *-
	Yes	5 (11.5)	1.87 (0.68, 5.13)	
Insomnia symptoms	No	71 (7.0)	1 (Reference)	-* *-* *-
	Yes	5 (5.3)	0.70 (0.26, 1.90)	
Underweight (BMI <18.5)	No	60 (6.6)	1 (Reference)	-* *-* *-
	Yes	15 (7.7)	1.18 (0.63, 2.24)	
Tobacco use status	Never, former	43 (5.7)	1 (Reference)	-* *-* *-
	Current	33 (8.9)	1.63 (0.98, 2.71)	
Physical activity	Medium, high	40 (6.6)	1 (Reference)	-* *-* *-
	Low	36 (7.0)	1.06 (0.64, 1.76)	
ADL/IADL	None	45 (6.2)	1 (Reference)	-* *-* *-
	One	23 (7.6)	1.23 (0.69, 2.19)	
	Two or more	8 (9.6)	1.65 (0.71, 3.82)	

*∗∗∗*P<0.001; *∗∗*P<0.01; *∗*P<0.05. (I) ADL=(Instrumental) Activities of Daily Living.

## Data Availability

Data from the IFLS-5 is available from RAND at http://www.rand.org/labor/FLS/IFLS.html.

## Additional Points

*Policy Impact*. This study describes for the first time sociodemographic and modifiable risk factors for cognitive functioning in Indonesia.* Practice Impact*. Agencies focusing on the promotion of cognitive functioning programmes in older adults should integrate modifiable risk factors such as having hypertension and being underweight as identified in this study.
